# Cellular Discrepancy of Platinum Complexes in Interfering with Mitochondrial DNA

**DOI:** 10.1021/acscentsci.4c01941

**Published:** 2025-01-24

**Authors:** Suxing Jin, Yafeng He, Chenyao Feng, Jian Yuan, Yan Guo, Zijian Guo, Xiaoyong Wang

**Affiliations:** † School of Food Science and Pharmaceutical Engineering, Nanjing Normal University, Nanjing 210023, P. R. China; ‡ State Key Laboratory of Pharmaceutical Biotechnology, School of Life Sciences, Nanjing University, Nanjing 210023, P. R. China; § State Key Laboratory of Coordination Chemistry, School of Chemistry and Chemical Engineering, Nanjing University, Nanjing 210023, P. R. China

## Abstract

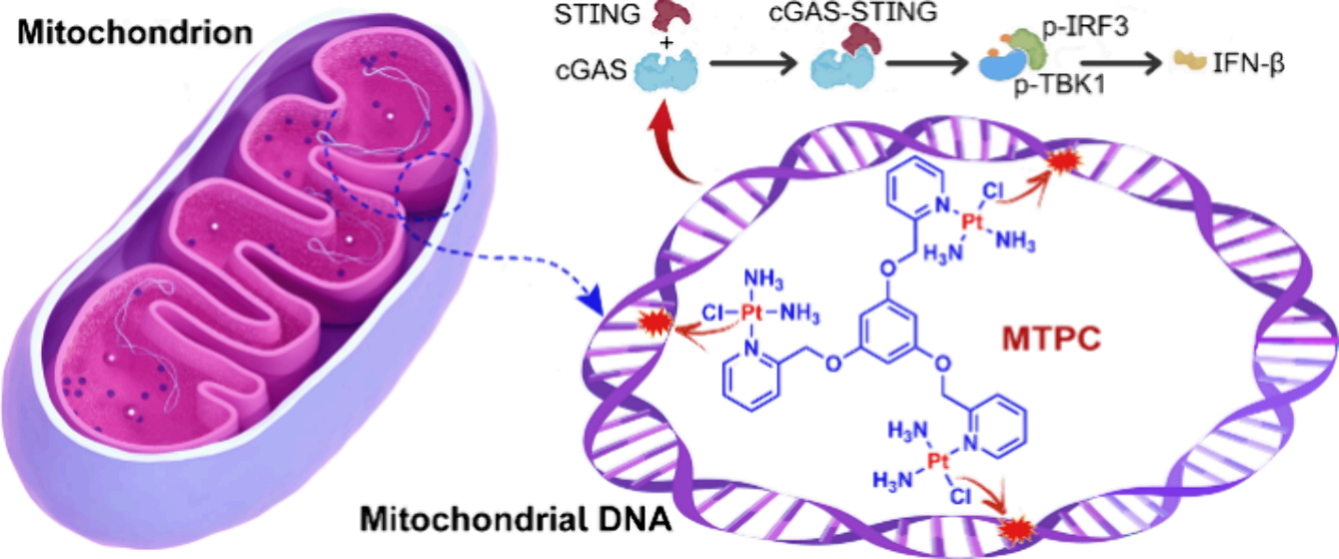

Mitochondria are associated with cellular energy metabolism, proliferation, and mode of death. Damage to mitochondrial DNA (mtDNA) greatly affects mitochondrial function by interfering with energy production and the signaling pathway. Monofunctional trinuclear platinum complex MTPC demonstrates different actions on the mtDNA of cancerous and normal cells. It severely impairs the integrity and function of mitochondria in the human lung cancer A549 cells, such as dissipating mitochondrial membrane potential, decreasing the copy number of mtDNA, interfering in nucleoid proteins and polymerase gamma gene, reducing adenosine triphosphate (ATP), and inducing mitophagy, whereas it barely affects the mtDNA of the human kidney 2 (HK-2) cells. Moreover, MTPC promotes the release of mtDNA into the cytosol and stimulates the cyclic GMP-AMP synthase-stimulator of interferon genes (cGAS-STING) pathway, thus showing the potential to trigger antitumor immunity. MTPC displays significant cytotoxicity against A549 cells, while it exhibits weak toxicity toward HK-2 cells, therefore displaying great advantage to overcome the lingering nephrotoxicity of platinum anticancer drugs. Discrepant effects of a metal complex on mitochondria of different cells mean that targeting mitochondria has special significance in cancer therapy.

## Abstract 2

Pt complex induces distinct mtDNA damage and death mode in different cell types, causes lesion to mitochondrial integrity and function, activates the cGAS-STING pathway, and reduces nephrotoxicity.

## Introduction

Platinum drugs have achieved tremendous success in the treatment of cancers, however, they also meet big challenges in the clinic due to high toxicity to normal tissues.^1,2^ Mitochondria have provoked extensive interest in the design of anticancer drugs owing to their important roles in many pathways essential to cell survival.^3,4^ About 80–90% of adenosine triphosphate (ATP) was generated by mitochondrial oxidative phosphorylation (OXPHOS), which is crucial for multiple cellular metabolisms and pro- or antiapoptotic stimulations in cancers.^5^ Cancer is characterized by altered energy metabolism involving not only abnormal gene expression in nuclear DNA (nDNA), but also mutations in mitochondrial DNA (mtDNA).^6,7^ MtDNA is almost completely comprised of sequences coding for 13 OXPHOS subunits, 22 tRNAs and 2 rRNAs. Noncoding mtDNA accounts for only less than 10%.^8,9^ The main noncoding region is located in the displacement (D)-loop, a 1.1 kb region controlling mtDNA replication and transcription.^10^ Alterations in mtDNA could cause severe consequences that are related to intracellular energy supply.^11^ Since mtDNA lacks histone protection and repair capacity, it is more vulnerable to anticancer agents.^12,13^ Moreover, the damaged mtDNA could be released to cytoplasm, where it is recognized by cyclic GMP-AMP synthase (cGAS) to activate the stimulator of interferon genes (STING), triggering both innate and adaptive immune responses.^14,15^

**Figure 1:**
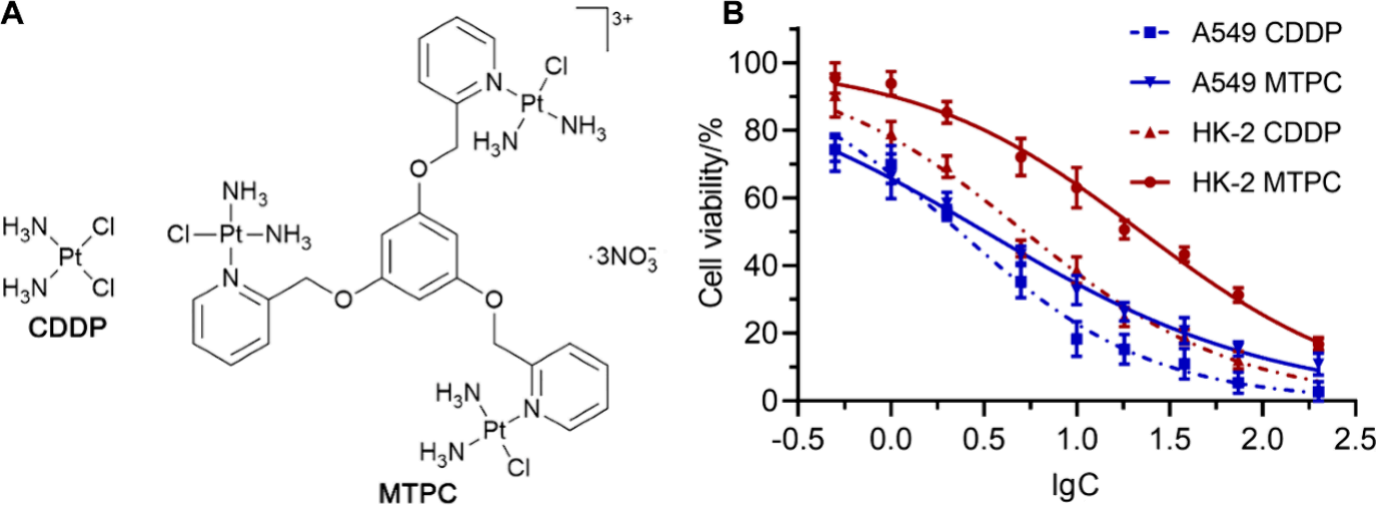
Chemical structures of CDDP and MTPC (A) and their cytotoxicity toward A549 and HK-2 cells after treatment for 48 h determined by the MTT assay. Data are presented as means ± standard deviation (S.D.; *n* = 3).

Traditional platinum anticancer drugs primarily target nDNA, resulting in cytotoxicity to cancerous and normal cells;^16^ however, many new platinum complexes were found to react with mtDNA, leading to cytotoxicity associated with the reduction of mtDNA copy number and activation of cGAS-STING pathway.^17,18^ Years ago we reported that a monofunctional trinuclear platinum complex MTPC (Figure 1A) formed long-range intra- and interstrand DNA adducts and exerted potent cytotoxicity against the human non-small-cell lung cancer (NSCLC) A549 cells.^19^ Nevertheless, the interaction details with DNA and the anticancer mechanism are not clear. Now we know that platinum complexes can interact with organelles, proteins, signal pathways, or mtDNA to exert cytotoxicity.^20^ Since MTPC has a tendency to accumulate in mitochondria due to its multiple positive charges and lipophilicity, we hence suppose that it may inhibit cancer cells through altering the mitochondrial genome in cancer cells.

Herein we investigate the cytotoxic activity and mechanism of MTPC toward cancer and normal cells through exploring its action on mitochondria and mtDNA. The results reveal that MTPC has greater impact on mtDNA of A549 cells, with its cytotoxicity positively correlating to the extent of mtDNA damage. The results indicate that MTPC is a special anticancer agent that shows different activities toward cancerous and normal cells and therefore could be a unique drug candidate for coping with the systemic toxicity of platinum drugs.

## Results and Discussion

### Cytotoxicity

NSCLC is the most common type of lung cancer and one of the leading causes of cancer death in the world.^21^ We have proved previously that MTPC is a potent anticancer agent in several cancer cell lines, including the NSCLC cell line A549.^19^ In this study, we retested its cytotoxicity against A549 cells by the MTT assay to ascertain its anticancer activity. Nephrotoxicity is the primary dose-limiting toxicity for cisplatin (CDDP), which causes kidney damage and impairs renal functions.^22^ Therefore, we also tested the effect of MTPC on human kidney 2 (HK-2) cells to assess its nephrotoxicity. As shown in Figure 1B and Table S1, the half maximal inhibitory concentrations (IC_50_) of MTPC seem somewhat weaker than the earlier report (3.0 vs 1.5 μM at 48 h) possibly because the cellular status was different. The cytotoxicity of MTPC is comparable to that of CDDP (ca. 2.4 μM) against the A549 cells; however, it is much weaker (>21 μM) than that of CDDP (ca. 5 μM) toward the HK-2 cells. Since most platinum anticancer agents have nephrotoxicity,^23^ and about 30% patients develop acute kidney injury or chronic kidney disease after CDDP administration,^24^ the relative low toxicity of MTPC toward renal cells is particularly meaningful for avoiding this detrimental effect.

### Cellular Distribution and DNA Platination

**Figure 2:**
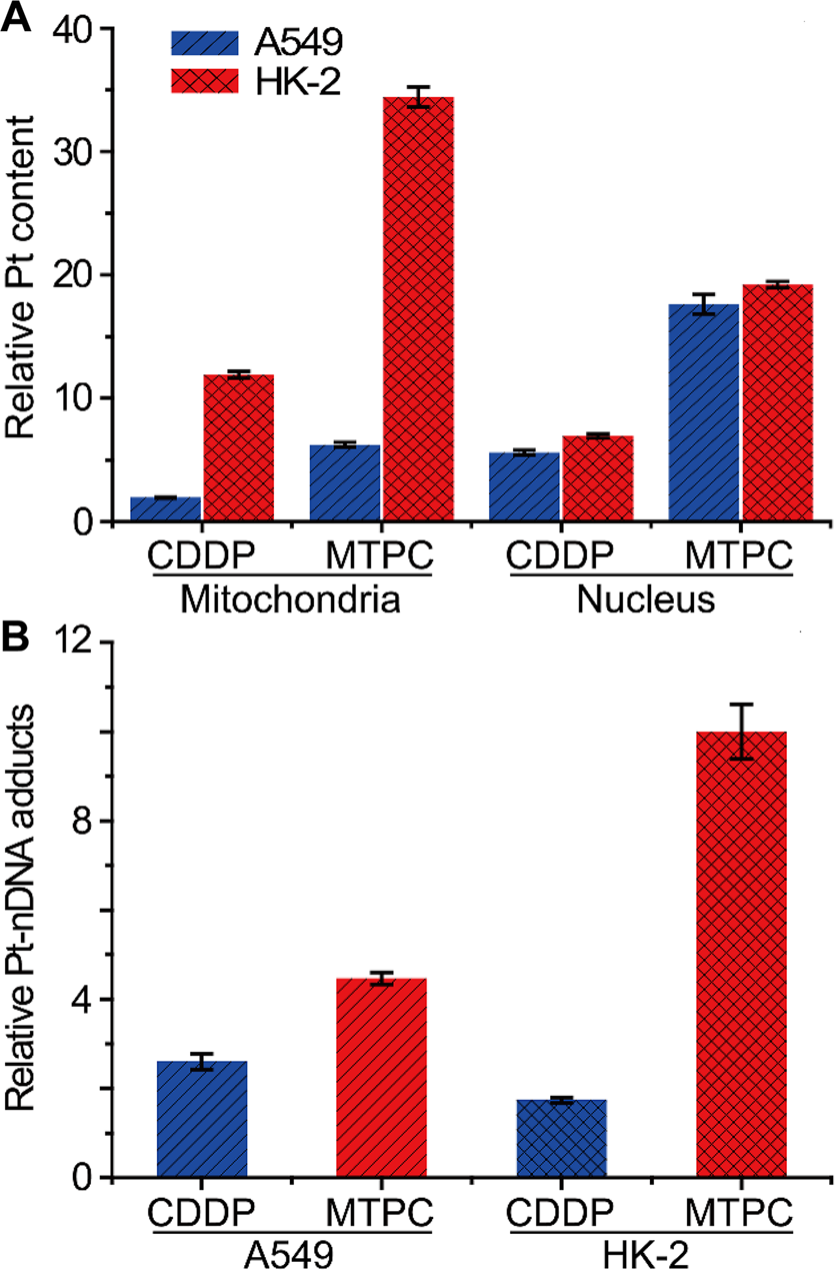
Subcellular distribution of MTPC and CDDP in A549 and HK-2 cells in terms of Pt determined by ICP-MS (A), and Pt-nDNA adducts quantified in cellular DNA extracted from A549 and HK-2 cells (B) after treatment with CDDP or MTPC (10 μM) for 12 h, respectively. Data are presented as means ± S.D. and normalized to the control.

Both mitochondria and the nucleus contain their own DNA and hence could be the targets of platinum complexes. In order to explore the potential mechanism for the differential cytotoxicity between MTPC and CDDP in cancerous and normal cells, we isolated the mitochondria and nuclei and quantified the Pt content in them using ICP-MS. As shown in Figure 2A, in A549 cells, MTPC and CDDP mainly accumulated in the nuclei; in HK-2 cells, MTPC and CDDP mainly accumulated in the mitochondria. However, considering that MTPC is a trinuclear complex, the accumulation of MTPC and CDDP in nuclei or mitochondria is almost the same in terms of molecule number. The results suggest that MTPC and CDDP did not exhibit obvious differences in subcellular selection. We further determined the amount of DNA-bound Pt in Pt-nDNA adducts in A549 and HK-2 cells. As shown in Figure 2B, in A549 cells, MTPC is less effective than CDDP in forming Pt-nDNA adducts in terms of each Pt atom. In other words, it is less effective in damaging nDNA than CDDP. Interestingly, in HK-2 cells, MTPC formed much more Pt-nDNA adducts than CDDP did although its cytotoxicity toward this cell line was lower than CDDP. Therefore, the cytotoxicity of platinum complexes is not necessarily only dependent on nDNA damage; other factors such as mtDNA damage, protein changes, and cell kind may also contribute to the difference of cytotoxicity. Evidently, the distinction of cytotoxicity between MTPC and CDDP against cancerous and normal cells cannot be explained by the common “high uptake, high toxic” notion.^25^

### MtDNA Damage and Transcriptional Inhibition

**Figure 3:**
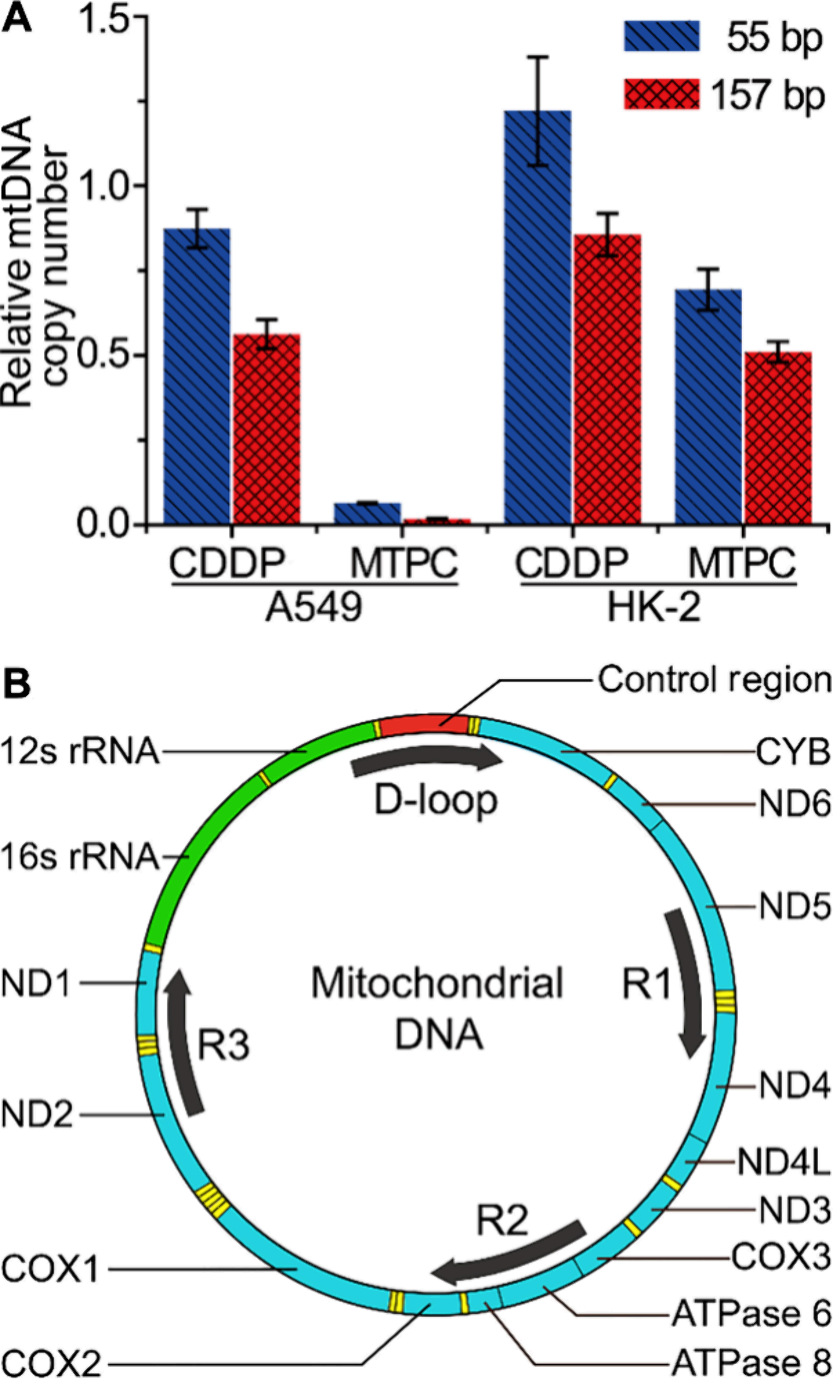
Copy number of mtDNA determined by amplification of two mtDNA fragments (55 and 157 bp) using an LC3 nuclear sequence in A549 and HK-2 cells treated with CDDP or MTPC (3 μM) (A), and illustration of the mitochondrial genome and qPCR amplified fragments (B). The analysis was based on the cross point Ct values for each fragment, and the copy number variation was calculated by the 2^–ΔΔCt^ method. Data are presented as means ± S.D.

Since the nDNA-binding data are inconsistent with the cytotoxicity of MTPC and CDDP against cancerous and normal cells, we further investigated the impact of MTPC and CDDP on mtDNA. The copy number of mtDNA is important for oxidative respiration and mitochondrial maintenance. The ratio of mtDNA to nDNA is often used to estimate the mtDNA copy number per cell, which ranges from several hundreds to more than 10,000 depending on the cell type.^9^ We determined the mtDNA copy number variations in A549 and HK-2 cells after treatment with MTPC or CDDP by monitoring the mtDNA content using real-time quantitative polymerase chain reaction (qPCR).^26^ As shown in Figure 3A, the mtDNA copy number decreased dramatically (>90% loss vs control) after treatment with MTPC in A549 cells, while CDDP only induced a slight or moderate loss. In HK-2 cells, the mtDNA copy number was only decreased about 50% by MTPC, while CDDP even raised the copy number to nearly 120% in comparison with the control. The results indicate that MTPC was more effective than CDDP in damaging mtDNA, especially in A549 cells. In other words, MTPC induced much less damage to mtDNA in HK-2 cells than that in A549 cells. The results derived from the two mtDNA sequences of different lengths (55 bp and 157 bp) are consistent with each other. Dramatic decline in mtDNA copy number would lead to a reduction of the mitochondrial components of the electron transport chain such as cytochrome c oxidase (COX), decreasing mitochondrial respiratory activity and contributing to membrane depolarization and production of reactive oxygen species (ROS).^27^

The damage to mtDNA was further verified by qPCR using four 1 kb DNA probes located in the mitochondrial genome as previously described.^26^ As shown in Figure 3B, four 1 kb-sized regions alternately distributed along mtDNA, where different colors indicate mitochondrial gene codes for 22 tRNAs (yellow), the 16s and 12s subunits of rRNA (green), 13 proteins comprising important complexes in the OXPHOS process (blue), and the replication origin for both heavy and light strands (red), respectively. Among them, three fragments (R1, R2, R3) seated in the regions containing the ND5/ND4 (primer R1), COX2/3/ATPase6/8 (primer R2), and ND1/ND2 (primer R3) genes, respectively. One amplicon (primer) was situated in the D-Loop, which exhibits a triple-stranded semistable DNA structure during replication in mtDNA and is supposed to be more prone to DNA damage agent than other regions due to its relatively relaxed structure. The method relies on the PCR-based amplification rate of two mtDNA fragments of different lengths. The amplification of the longer fragment serves as an experimental probe to assess the extent of damage induced by the complex. The adjacent shorter fragment (<90 bp) serves as an internal normalization control. Under physiologically relevant conditions, the amplification of shorter DNA fragments represents undamaged mtDNA because of less platinum attack. When mtDNA is damaged to a detectable extent, a delayed cross point (Ct) could be obtained. The change in amplification rate of mtDNA from the MTPC- or CDDP-treated cells was expressed as 2^–ΔΔCt^.^28^ The results are listed in Table 1. MTPC induced severe mtDNA damage in A549 cells, while CDDP only induced insignificant mtDNA damage, thus confirming MTPC is a potent mtDNA-damaging agent for A549 cells. In all the four regions, the mtDNA damage extent induced by MTPC is at least 1 order of magnitude higher than that induced by CDDP. Especially in the D-loop, the amplification rate of the MTPC-treated samples was reduced by 3 orders of magnitude compared to that of the CDDP-treated samples, which may be due to the relaxed structure of D-Loop facilitating the binding of MTPC to mtDNA. The D-loop locates in the main noncoding area of mtDNA, a segment known as the control region, where mtDNA replication and transcription regulation occur. Damage to this segment could directly affect mtDNA metabolism.^29^

**Table 1:** Quantification of mtDNA Lesion in A549 and HK-2 Cells after Incubation with MTPC and CDDP (3 μM), respectively, for 24 h Measured by qPCR

	A549		HK-2	
Region	MTPC	CDDP	MTPC	CDDP
D-loop	3268.2 ± 1254.6^a^	2.60 ± 0.95	0.57 ± 0.10	1.12 ± 0.33
R1	22.2 ± 0.9	1.48 ± 0.23	2.04 ± 0.42	0.98 ± 0.22
R2	16.6 ± 1.0	1.26 ± 0.10	0.85 ± 0.16	0.63 ± 0.08
R3	10.0 ± 1.2	0.61 ± 0.08	2.32 ± 0.70	3.50 ± 1.20

a Relative mtDNA damage extent is evaluated on the amplification efficiency alteration calculated by 2^–ΔΔCt^ method. Data are represented as means ± S.D.

Surprisingly, MTPC did not damage mtDNA severely in HK-2 cells, although it mainly accumulated in mitochondria (see Figure 2A). This is because the D-loop region is particularly susceptible to DNA mutations and mutant mtDNA in tumor cells is 19–220 times as abundant as mutated nDNA,^9,30^ whereas the mutations of mtDNA in healthy cells are very low.^31^ In A549 cells, MTPC was much more effective than CDDP in damaging mtDNA although it was less effective in damaging nDNA; in HK-2 cells, MTPC largely accumulated in the mitochondria but did not affect mtDNA significantly. Therefore, the distinct cytotoxicities of MTPC toward A549 and HK-2 cells could be attributed to its differentiated impacts on mtDNA with different mutations. The cytotoxicity of CDDP mainly arose from nDNA damage, which is similar for both the A549 and HK-2 cells.

### Binding Mode of MTPC with MtDNA

**Figure 4:**
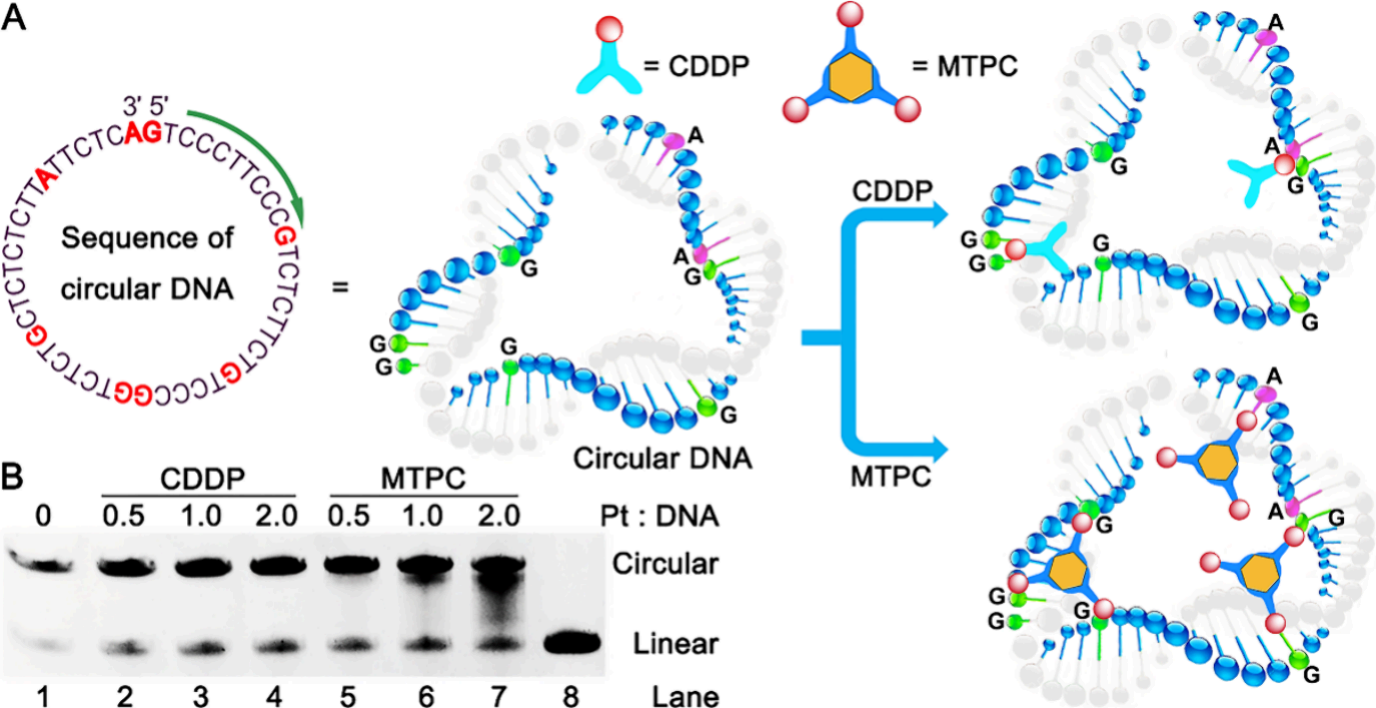
Conjectural binding of CDDP and MTPC to a model of 48 nt circular ssDNA with sparse guanines and adenines (A), and polyacrylamide gel electrophoresis patterns of 48 nt circular DNA after incubation with CDDP and MTPC respectively at 37 °C for 24 h. Lane 1, circular DNA control; lanes 2–7, [complex]/[DNA] = 0.5, 1.0, 2.0, respectively; lane 8, linear DNA control (B).

As the only circular DNA in cells, mtDNA has a unique chemical structure. Crystallographic studies have revealed that mitochondrial transcription factor A (mtTFA) induces a dramatic U-turn of an overall bend of 180° upon binding to mtDNA.^32^ Moreover, the nucleotides in mtDNA is more exposed and vulnerable than those in nDNA due to the lack of histone protection and nucleotide excision repair pathway.^33^ In order to study the different binding modes of CDDP and MTPC with mtDNA, a small circular DNA containing only one pair of adjacent GG and AG and several separate G or A with long distance intervals was constructed. As shown in Figure 4A, the circular DNA is a mimic of the moderate length stretches of the ssDNA loop created during mtDNA replication or arisen from mtDNA lesion. According to the structural features, CDDP is supposed to form 1,2-GG and 1,2-AG adducts due to its bond angle limitation;^34^ while MTPC tends to form long-range GG cross-links or monofunctional adducts with mtDNA. This speculation was supported by the result of polyacrylamide gel electrophoresis. As shown in Figure 4B, the gel band of circular DNA was barely affected by CDDP (lanes 2–4), suggesting that the formation of 1,2-GG and 1,2-AG adducts did not exert significant influence on the configuration of circular DNA. By contrast, the migration rate of circular DNA band was markedly accelerated (lanes 6 and 7) by high concentration of MTPC (≥1 μM), implying that the configuration of mtDNA was dramatically condensed or compressed due to the formation of some long-range DNA cross-links or greater electrostatic interaction between MTPC and circular DNA. The results show that MTPC is a more effective binder of mtDNA because of its trident structure and high positive charge (+3), which may lead to more severe damage to mtDNA and difference in cytotoxicity between MTPC and CDDP. On the other side, since MTPC could promote the generation of mitochondrial ROS (Figure S1), oxidative damage to mtDNA cannot be excluded, which has been verified by the DNA oxidative damage biomarker 8-hydroxy-2′-deoxyguanosine (8-OHdG, Figure S2).

### Impact on MtDNA–Protein Complex

**Figure 5:**
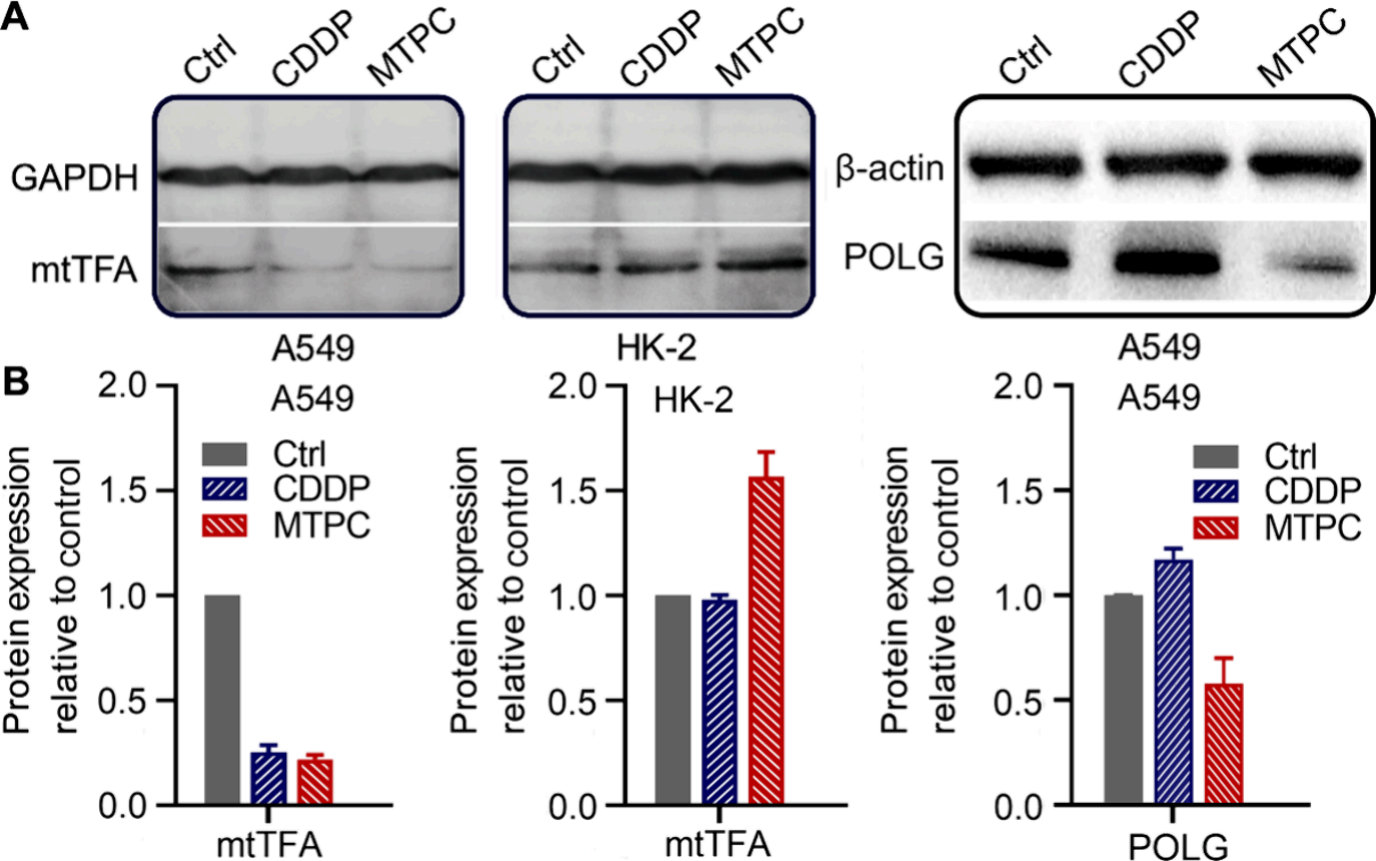
Expressions of mtTFA and POLG in A549 or HK-2 cells stimulated by CDDP or MTPC (3 μM) respectively for 24 h (A) and corresponding protein contents normalized to the control (B). Data are presented as means ± S.D.

With no chromosome-like structure, mitochondrial genome is organized into nucleoids, which are associated with the mitochondrial inner membrane and often wrapped around cristae.^35^ Nucleoids are DNA–protein complexes formed with the help of mtTFA, which is a high mobility group (HMG)-box containing protein with functions of packaging mtDNA, replication and transcription,^36^ and also a key regulator of mtDNA copy number.^37^ Therefore, mtTFA could be an index of mtDNA damage. The expression of mtTFA in A549 and HK-2 cells was detected by Western blotting after treatment with MTPC and CDDP, respectively. As shown in Figure 5, MTPC and CDDP remarkably reduced the expression of mtTFA in A549 cells; on the contrary, MTPC increased the expression of mtTFA in HK-2 cells. MtTFA binds to mtDNA, wraps around it completely, and favors the assembly of other proteins to compact mtDNA.^38^ The decrease of mtTFA in A549 cells suggests that mtDNA was more vulnerable to the attack of MTPC. Conversely, the increase of mtTFA in HK-2 cells implies that the mtDNA was well-protected and could not easily be damaged. Therefore, MTPC showed significant cytotoxicity against the former but less toxicity toward the latter.

In addition to mtTFA, polymerase gamma (POLG) is also a critical enzyme responsible for the replication, transcription and structural integrity of mtDNA.^39^ The interaction and feedback between POLG and mtTFA decide mtDNA homeostasis in various types of cells.^40^ We hence investigated the effect of MTPC on POLG in A549 cells after treatment for 24 h. As shown in Figure 5, the expression of POLG was significantly downregulated by MTPC, suggesting that the replication and repair of mtDNA were largely suppressed after the damage, thus leading to the decrease of mtDNA copy number. CDDP did not affect POLG obviously, indicating that its action on mtDNA was quite weak. Taken together, MTPC not only damaged mtDNA, but also influenced the expression of proteins relevant to the integrity, replication, and transcription of mtDNA in A549 cells.

### Mitochondrial Dysfunction

**Figure 6:**
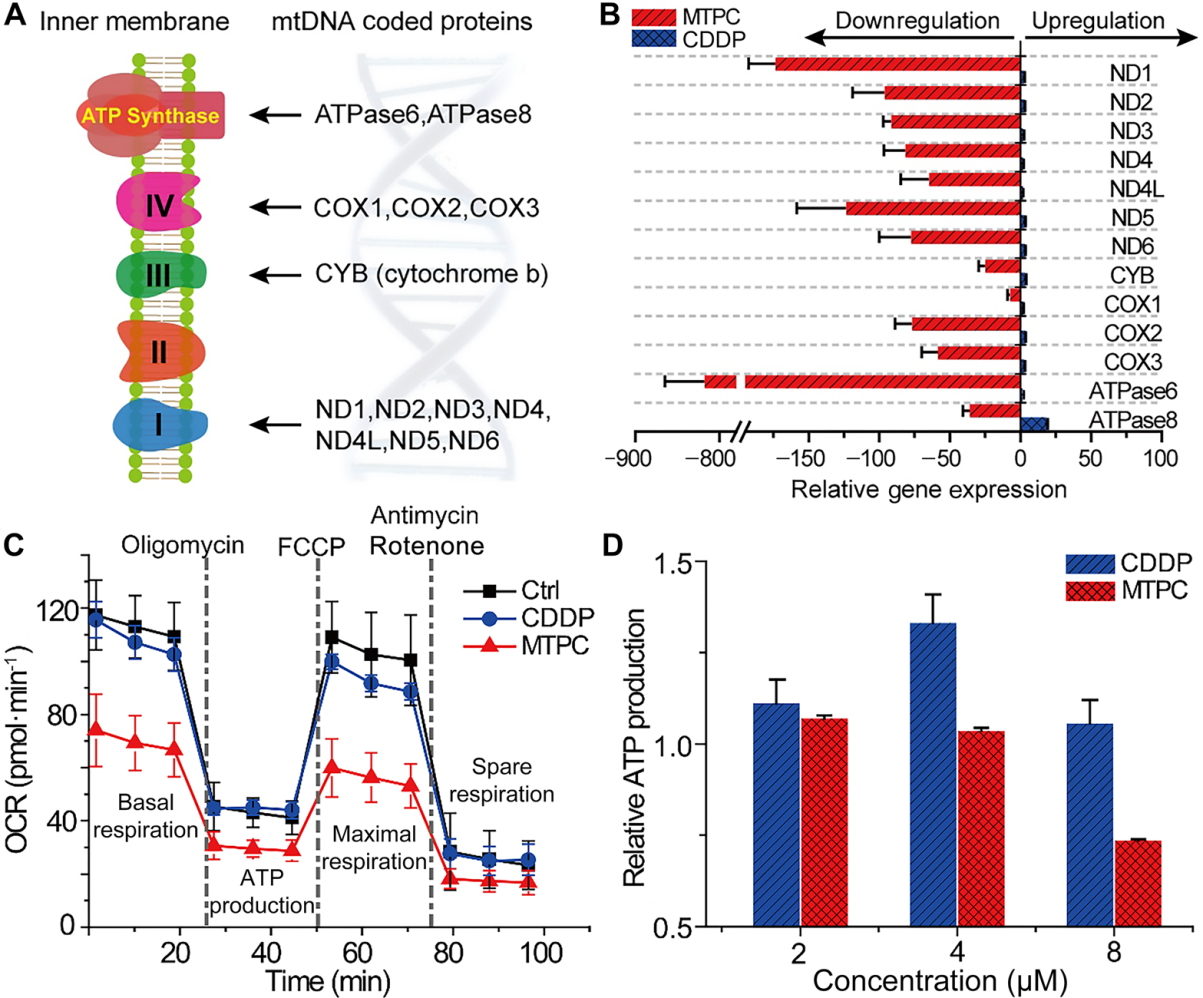
Proteins encoded by mtDNA (A), mitochondrial gene expression profile (B), mitochondrial bioenergetic profiles (C), and ATP production (D) of A549 cells treated with MTPC and CDDP (3 μM) respectively, or 2, 4, and 8 μM for 24 h. The relative gene expression level is evaluated by reverse transcription-q PCR (RT-qPCR), normalized to GAPDH and the control; data are means of 3 parallel experimental results.

Mitochondrial genomic aberration and deficiency in mtTFA and POLG can decrease the mtDNA copy number, leading to the disorder of respiratory chain and mitochondrial dysfunction.^41^ The function of mitochondria in bioenergetics relies on the OXPHOS system consisting of five protein complexes (I–IV and ATPase), which are partly encoded by mtDNA except for complex II (Figure 6A).^9,35^ The core complexes of oxidation respiratory chain are constituted by 13 mitochondrial genome coded proteins, including ND1–6, ND4L, CYB, COX1–3, ATPase6 and ATPase8.^9^ The above results show that MTPC induced severe damage to mtDNA in A549 cells; therefore, it may affect the oxidative respiration of mitochondria, leading to mitochondrial dysfunction. The downstream consequences of mtDNA damage were investigated using qPCR. As shown in Figure 6B, the mRNA expressions of all the 13 genes in the MTPC-treated A549 cells were downregulated at least 25 times except COX1. Specifically, the gene expressions of ND1–6 and ND4L decreased over 50 times, and ATPase6 decreased over 800 times, indicating that the ATP production in mitochondria would be significantly inhibited by MTPC. On the other hand, CDDP slightly upregulated the expression of the 13 genes to meet the higher energy need by the cellular defense system. These results indicate that MTPC and CDDP induced opposite mitochondrial gene expressions in cancer cells. By damaging mtDNA, MTPC silenced the gene expression of key components in the OXPHOS system, disrupted the oxidation respiratory chain, and thereby impaired the function of mitochondria.

The main function of mitochondria is to produce ATP. To determine the impact of MTPC on mitochondrial OXPHOS, the respiratory capacity of A549 cells in the presence of MTPC was investigated on a Seahorse XF^e^24 bioanalyzer. As shown in Figure 6C, the basal oxygen consumption rate (OCR, an index of OXPHOS) of the MTPC-treated A549 cells was significantly lower than that of the CDDP-treated or control cells, indicating that MTPC reduced the basal metabolic activity of mitochondria. Addition of oligomycin (an ATP synthase inhibitor) reduced the OCR more evidently in the MTPC-treated cells than in the CDDP-treated or control cells, suggesting that MTPC inhibited the ATP synthetic capability. After the addition of carbonyl cyanide p-trifluoromethoxyphenylhydrazone (FCCP, a mitochondrial uncoupler), the maximal respiration decreased markedly for the MTPC-treated cells, showing that MTPC impaired the respiratory function of mitochondria. Subsequent addition of antimycin and rotenone (electron transport chain inhibitors) significantly lowered the spare respiratory capacity of the MTPC-treated cells, suggesting that MTPC diminished the adaptability or flexibility of mitochondria. CDDP only mildly affected respiratory function. The inhibition of ATP synthetic ability by MTPC was also confirmed by the luciferase-based ATP assay kit.^42^ As shown in Figure 6D, the production of ATP was reduced in the MTPC-treated A549 cells in a concentration-dependent manner, indicating that the mitochondrial function was severely damaged; whereas CDDP elevated the ATP level in A549 cells. These results demonstrate that MTPC inhibited the mitochondrial OXPHOS and ATP production ability of A549 cells, which are consistent with the gene expression profile in Figure 6B, where ATPase6 was prominently downregulated by MTPC. All in all, MTPC downregulated the mitochondrial gene expression, undermined mitochondrial respiration, and inhibited the production of ATP in A549 cells, thereby showing a distinct cytotoxicity from CDDP.

### Mitochondrial Morphology and Cell Death Mode

**Figure 7:**
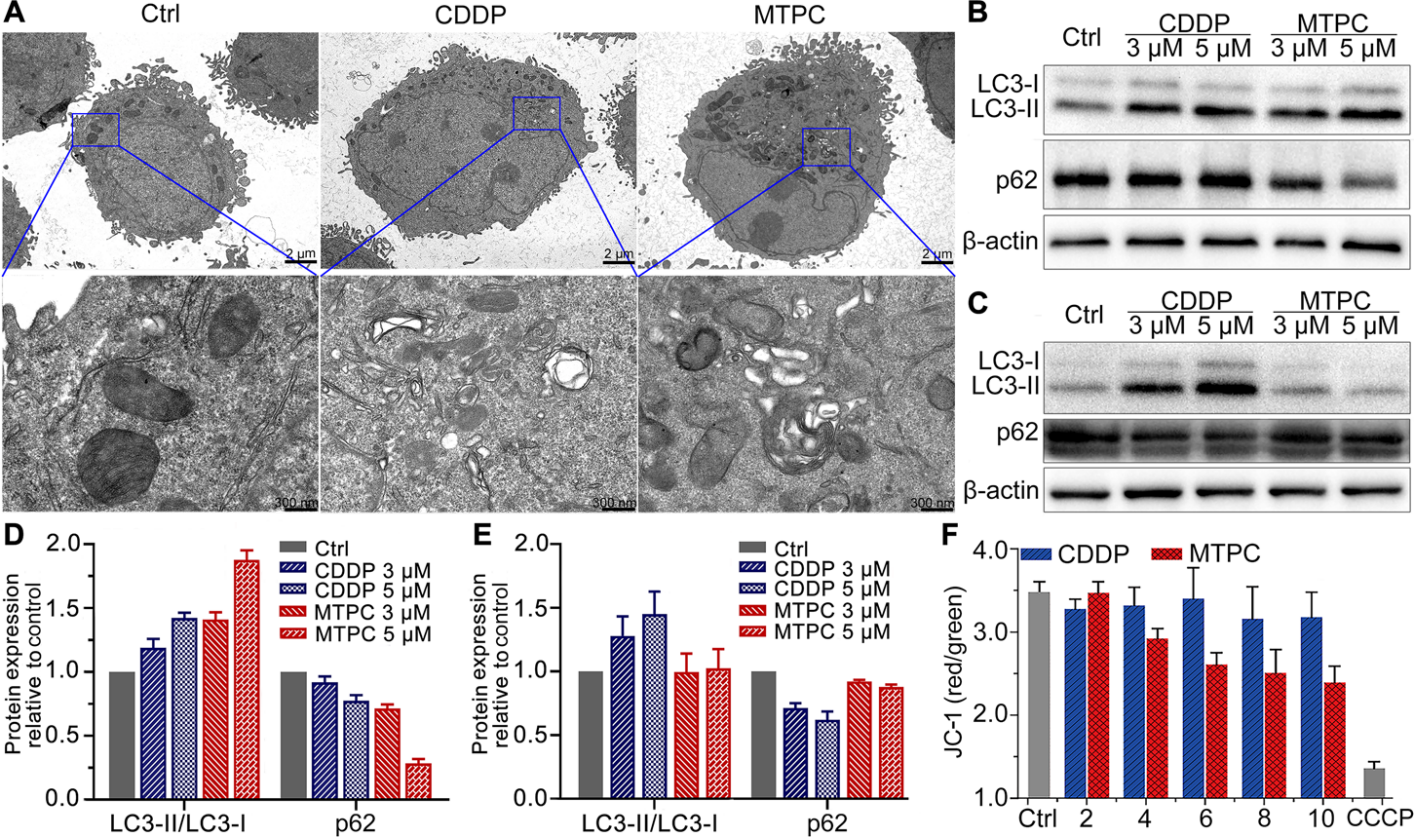
TEM images of mitochondria in A549 cells after treatment with each complex (3 μM) for 24 h (A), expression and corresponding protein contents of LC3-II/I and p62 relative to the control in A549 (B, D) and HK-2 (C, E) cells after incubation with each complex (3 μM) for 24 h determined by Western blotting, and relative fluorescence ratios of JC-1_red_ to JC-1_green_ in A549 cells after treatment with different concentrations of MTPC and CDDP for 24 h, respectively (F). Data are presented as means ± S.D.

Previously, we have shown that MTPC induced apoptosis in cancer cells;^19^ the above evidence indicates that it also has a great impact on mtDNA and mitochondrial function. Therefore, we speculate that MTPC may alter the overall structure of mitochondria and further alter the cell death mode. The effect of MTPC on mitochondrial morphology was investigated by using transmission electron microscopy (TEM). As shown in Figure 7A, in the untreated A549 cells, mitochondria took the normal shape with clear and intact cristae; while in the MTPC-treated cells intact mitochondria were rarely seen; instead, the fusion of mitochondria and lysosomes, mitophagic vacuoles, or mitophagosomes containing damaged mitochondria were observed. The observation suggests that MTPC seriously damaged the structure of mitochondria and induced mitophagy in A549 cells,^43^ which removes defective mitochondria to prevent mitochondrial accumulation and deterioration.^44^ The influence of CDDP on mitochondria was weaker than that of MTPC. The mitophagy induced by MTPC was further confirmed by Western blotting. The conversion of microtubule-associated protein 1 light chain 3 (LC3) from LC3-I to LC3-II reflects the progression of mitophagy and the amount of LC3-II correlates with the number of mitophagosomes.^45^ As shown in Figure 7B and D, in A549 cells, MTPC upregulated the active LC3-II membranous protein to drive the membrane alterations required for mitophagosome formation; meanwhile, it downregulated protein p62, which is another marker of mitophagy. The fusion of mitochondria with lysosomes results in the formation of autolysosomes and the degradation of autophagic protein p62; therefore, when mitophagy occurs, the amount of p62 decreases.^45^ CDDP also elevated the expression of LC3-II obviously but did not downregulate p62, indicating that it only induced a mild mitophagic response, which is consistent with the TEM image. Interestingly, the Western blots in Figure 7C and E show that MTPC did not induce mitophagy in HK-2 cells, whereas CDDP did, which explains the higher cytotoxicity of CDDP toward the normal HK-2 cells. MtDNA damage can trigger the selective removal of dysfunctional mitochondria. Mitophagy is important in response to mtDNA damage, serving as a clearance mechanism when mtDNA damage overwhelms repair mechanisms. Unrepaired mtDNA damage is cleared either via direct degradation or via mitophagy.^46^ Since MTPC impaired more mtDNA in A549 cells than in HK-2 cells, it induced more significant mitophagy there.

In order to further estimate the impact of MTPC on the mitochondrial integrity, mitochondrial membrane potential (ΔΨ_m_) in A549 cells was detected using fluorescence probe JC-1 (5,5,6,6′-tetrachloro-1,1′,3,3′-tetraethyl-imidacarbocyanine iodide), whose fluorescence shifts from red to green as the mitochondrial membrane is damaged and ΔΨ_m_ is reduced.^47^ As shown in Figure 7F, the ratio of JC-1_red_ to JC-1_green_ decreased obviously in the MTPC-treated cells as compared with the control, suggesting that MTPC enhanced mitochondrial permeability and reduced ΔΨ_m_. By contrast, CDDP barely affected ΔΨ_m_ under the same condition. The results show that the mitochondrial integrity of A549 cells was impaired by MTPC. Mitochondrial membrane depolarization also promotes mitophagy.^48^

### Activation of the cGAS-STING Pathway

**Figure 8:**
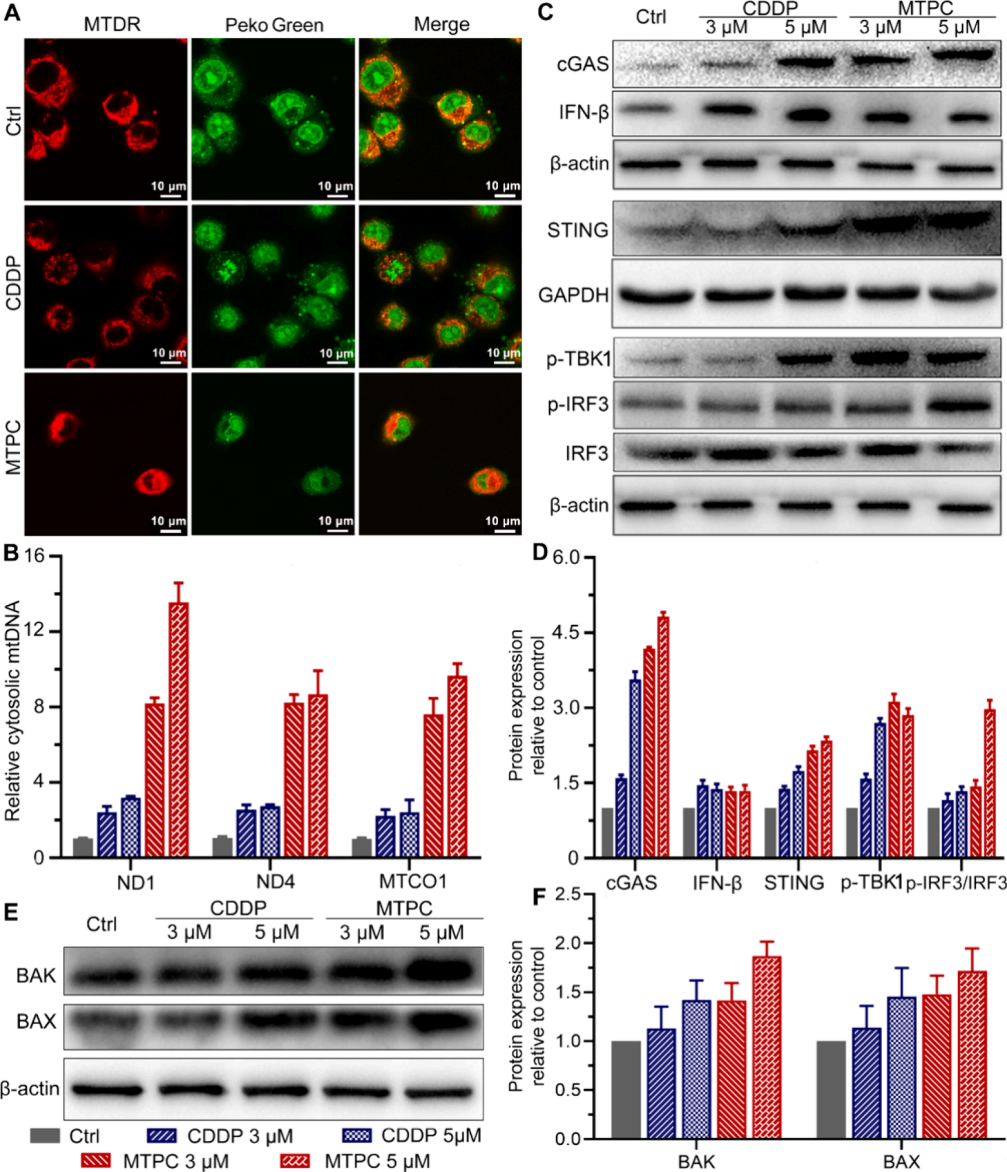
Fluorescence images of CDDP- and MTPC-treated A549 cells stained with MitoTracker (red)/Peko Green (green) reflecting the release of mtDNA (A), levels of ND1, ND4 and MTCO1 genes determined by qPCR (B), expression of cGAS, STING, IFN-β, p-TBK1, IRF3, p-IRF3, BAK, BAX (C, E), and corresponding protein content relative to control (D, F) in A549 cells after treatment with MTPC or CDDP for 24 h.

The cGAS-STING signaling pathway exerts regulatory functions in antitumor immunity through inducing cytokines, primarily Type I interferons (IFN-I).^49^ cGAS binds to double-stranded DNA (dsDNA) and activates the downstream STING and TANK-binding kinase 1 (TBK1) to induce the phosphorylation and nuclear trafficking of IFN regulatory factor 3 (IRF3), triggering the production of IFN-I and other cytokines for resetting antitumor immunity and promoting tumor eradication.^50^ MTPC caused severe damage to mtDNA and mitochondrial membrane, the damaged mtDNA may release into cytoplasm to activate the cGAS.^51,52^ Moreover, the reduction of mtTFA and POLG in cancer cells could lead to mitochondrial dysfunction and mtDNA cytosolic leakage, resulting in the activation of cGAS-STING pathway.^53,54^ The release of mtDNA into the cytoplasm of A549 cells was detected by a mitochondrion-specific fluorescent dye MitoTracker Deep Red (MTDR) and a dsDNA fluorescent probe Peko Green.^18^ As shown in Figure 8A, the red fluorescence indicates the region where mitochondria appear. The green fluorescence in the control appeared in the mitochondria and nuclei because both of them contain dsDNA. After treatment with CDDP, the green fluorescence decreased and dispersed, suggesting that some nDNA and mtDNA were damaged by CDDP and released into the cytoplasm. In the MTPC-treated cells, the green fluorescence mainly appeared in nuclei, while that in mitochondria almost disappeared completely, implying that MTPC only moderately affected nDNA but severely damaged mtDNA and mitochondrial membrane to release the mtDNA fragments into the cytoplasm. To further confirm the release of mtDNA, the cytosol was separated from the mitochondrial fraction and the level of specific mtDNA genes was determined by qPCR. As shown in Figure 8B, MTPC significantly increased the level of ND1, ND4 and MTCO1 (a protein coding gene that encodes cytochrome c oxidase subunit 1) genes, suggesting a mass of mtDNA was accumulated in the cytosol. By contrast, CDDP only slightly increased the number of mtDNA genes in the cytosol. The results indicate that MTPC damaged mtDNA and promoted its release into the cytoplasm.

The impact of MTPC on the cGAS-STING pathway in A549 cells was investigated by Western blotting. As shown in Figure 8C and D, the expression of cGAS and STING was significantly upregulated by MTPC in a concentration-dependent manner. Moreover, phosphorylated TBK1 (p-TBK1), IRF3, p-IRF3 and IFN-β were upregulated as well, which manifests that MTPC activated the cGAS-STING pathway in A549 cells. CDDP also activated the cGAS-STING pathway at a relatively higher concentration (5 μM), but the activation was mainly triggered by nDNA rather than mtDNA. The results demonstrate that MTPC is a more effective agonist of the cGAS-STING pathway than CDDP due to its more effective damage to mtDNA.

Our previous study has proved that MTPC can induce apoptosis,^19^ suggesting that the proapoptotic proteins BAK and BAX may be activated. Activation of BAK and BAX could result in the formation of large macropores in the mitochondrial outer membrane, which allow the inner mitochondrial membrane to herniate into the cytosol and release the mtDNA into the cytoplasm.^55^ The impact of MTPC on BAK and BAX in A549 cells was thus evaluated by Western blotting. As shown in Figure 8E and F, MTPC upregulated the expressions of BAK and BAX, hence could enhance the permeabilization of outer mitochondrial membrane. The results account for the escape of damaged mtDNA into the cytoplasm and the activation of cGAS/STING pathway in A549 cells.^56^

## Conclusion

Platinum complexes are potential anticancer drugs with nDNA as the primary target. However, they often cause severe damage to normal cells due to a lack of selectivity for cancer cells. Fortunately, the mitochondrial structure, function, and molecular composition of the inner membrane are different between cancerous and normal cells. These differences offer the opportunity to design anticancer drugs with mitochondria as the site-specific target. More importantly, the mtDNA sequence and copy number of cancerous cells are different from those of normal cells, subtle changes in the mitochondrial genotype can have profound effects on the nucleus and cancer progression.^30^ In recent years, a variety of mitochondrion-targeting compounds have exhibited anticancer activities in vitro and in vivo, but their advantages in overcoming systemic toxicity have not been explored. In this study we demonstrated that MTPC induces far more mtDNA lesions in A549 cancerous cells than that in HK-2 normal cells, hence displaying less toxicity toward the latter. Moreover, MTPC disrupts the mitochondrial energy metabolism, impedes the mitochondrial respiratory chain, and prompts the release of mtDNA fragments into the cytoplasm to activate the cGAS-STING pathway in A549 cells, thus showing the potential to stimulate antitumor immune responses.

Nephrotoxicity is a major adverse effect of CDDP that limits its long-term use in the treatment of NSCLC.^57^ The toxic mechanism has been related to the drug metabolic pathways and genomic risk factors,^58,59^ including DNA damage and apoptotic pathways. Currently, the CDDP-induced nephrotoxicity is chiefly prevented by hydration.^60^ MTPC selectively impairs mtDNA and induces mitophagy in A549 cancer cells but not in renal cells, which provides a basis for selective cancer cell killing without harming normal cells. MtDNA heteroplasmy is remarkably common among different cells; and the level of heteroplasmy can vary between cells from organ to organ within the same person.^9^ In view of the big differences in mtDNA of cancerous and normal cells, intervening in mtDNA may represent an effective strategy for enhancing the selectivity and improving the safety of platinum anticancer drugs.

## Data Availability

The authors confirm that the data supporting this article have been included within the article and as part of the Supporting Information.

## Supporting Information Available

Reagents and apparatus, experimental details, supplementary figures and tables, and the primer sequences used in experiment (PDF)
