# Supplementary material for: Cellular Discrepancy of Platinum Complexes in Interfering with Mitochondrial DNA
Source: ACS Cent Sci. 2025 Jan 24;11(3):393–403. doi: 10.1021/acscentsci.4c01941 (PMC11950849; doi:10.1021/acscentsci.4c01941)
Supplement: Supplementary file 1 — oc4c01941_si_001.pdf [file oc4c01941_si_001.pdf]

Supporting Information

Cellular Discrepancy of Platinum Complexes in Interfering with Mitochondrial DNA

Suxing Jin,[#] Yafeng He,[#] Chenyao Feng, Jian Yuan, Yan Guo, Zijian Guo,* and Xiaoyong Wang*

1. Experimental Details

Materials and Methods

All the reagents and solvents were of analytical grade and used as received without further purification. CDDP was purchased from Shandong Boyuan Pharmaceutical Co., Ltd. China. MTPC was prepared as previously reported.[1] 3-(4,5-Dimethylthiazol-2-yl)-2,5-diphenyltetrazolium bromide (MTT) was purchased from Sigma-Aldrich. Hydrogen peroxide (30%), concentrated hydrochloric acid and nitric acid were purchased from J&K Scientific. Genomic DNA mini preparation kit and ReadiPrep™ mitochondrial/cytoplasmic fractionation kit were purchased from Tiangen Biotech (Beijing) Co., Ltd. ATP assay kit, enhanced mitochondrial membrane potential assay kit with JC-1, mitochondria and nuclei isolation kit, DAPI staining solution and Mito-Tracker Deep Red FM were purchased from Beyotime Biotechnology (Shanghai, China). TRIzol reagent was purchased from Life Technologies. MitoTracker™ for mitochondrion labeling was purchased from Invitrogen Co. (Thermo Fisher Scientific). Peko Green for dsDNA was purchased from AAT Bioquest Inc. The antibodies for BAK (29552-1-AP), BAX (50599-2-Ig), LC3B (18725-1-AP), and p62 (18420-1-AP) were purchased from Proteintech Group, Inc. (Wuhan, China); DNA Polymerase γ (D1Y6R) (13609T) and 8-OHdG (DNA/RNA Damage) were purchased from Cell Signaling Technology, Inc. and MedChemExpress LLC, respectively; those for mtTFA (ab307302), cGAS (ab302617), IFN-β (ab171081), STING (ab239074), p-TBK (ab109272), p-IRF3 (ab320082), IRF3 (ab68481), GAPDH (ab181602) and β-actin

(ab213262) were purchased from Abcam. Water was deionized and ultrafiltered on a Milli-Q apparatus (Millipore Corporation, China). The SsoFast EvaGreen Supermix (Bio-Rad) was used according to the manufacturer's protocol.

The optical density (OD) of formazan was determined on a Varioskan flash multimode reader. Platinum content was analyzed on an ELAN 9000 inductively coupled plasma-mass spectrometer (ICP-MS, PerkinElmer Inc.). Western blotting was carried on the Bio-Rad Mini-PROTEAN Tetra system and Bio-Rad Powerpack Universal. Transmission electron microscope (TEM) images were taken by the Hitachi TEM system. Real-time quantitative polymerase chain reaction (qPCR) and reverse transcription-q PCR (RT-qPCR) was performed on a CFX Connect^{TM} Real-Time System (BIO-RAD, USA).

Cell Culture

The human non-small-cell lung cancer (A549) and human kidney 2 (HK-2) cells were purchased from the American type culture collection (ATCC). A549 cells were cultured in Roswell Park Memorial Institute 1640 medium (RPMI, KeyGEN BioTECH) with 10% fetal bovine serum (FBS), while HK-2 cells were cultured in DMEM/F12 medium (KeyGEN BioTECH) with 12% FBS at 37 ºC in a humidified atmosphere containing 5% CO_2. Appropriate penicillin and streptomycin were used as supplements.

Cytotoxicity

A549 or HK-2 cells were seeded in a standard 96-well plate with different densities (4×10^3–1×10^4 cells per well) and allowed to adhere and proliferate for 24 h in the medium prior to addition of the complex. CDDP stock solution (2 mM) was prepared in PBS, and MTPC stock solution (20 mM) was prepared in DMSO. The stock solution of CDDP or MTPC was diluted in complete medium (DMSO concentration < 5‰) to different concentrations and added in aliquots of 200 μL per well. After exposure for 48 h, the cells were treated with MTT (20 μL, 5 mg mL^{-1} in PBS) for 4 h. The medium was discarded and DMSO (150 μL) was added. The plates were vibrated at room temperature for 15 min and the absorbance of the solution was measured on a Varioskan flash multimode reader at 570 nm.

Cellular Distribution and DNA Platination

Platinum uptake was assessed as described previously.[2] Cells were inoculated in a 6-well plate at a density of 2×10^5 cells per well to preincubate for 24 h, and then treated with the complex (10 μM) for 12 h. The cells were harvested and washed twice with PBS. Mitochondria and cytoplasm in the cells were isolated using the mitochondria/cytosol fractionation kit. The concentration of cytosol protein was determined using the protein assay kit (Bio-Rad, Hercules, CA, USA). The cell pellets were digested by nitric acid (100 μL, 2 h), H_2O_2 (30%, 50 μL, 1.5 h) and HCl (100 μL, 2 h) at 95 °C, respectively. The solution was homogenized and diluted with water to determine the Pt content by ICP-MS. Cellular genomic DNA was extracted and quantified with Nanodrop 1000 at 260 nm. The Pt content in DNA was determined by ICP-MS.

Synthesis of Circular DNA

Short ssDNA B-C and B-sp (Table S2) were added to the reaction buffer consisting of $MgCl_2$ (6.6 mM), DTT (10 mM), ATP (0.1 mM) and Tris-HCl (66 mM, pH 7.6) at final concentrations of 3.5 and 4.5 μM, respectively. The solution was heated at 95 °C for 5 min, followed by addition of T4 ligase (10 μL) and incubation at 16 °C for 16 h. The enzyme was inactivated by incubation at 65 °C for 10 min. The crude cyclic DNA B-C was obtained after incubation at 37 °C for 30 min with the addition of exonuclease (15 μL). The purified cyclic DNA B-C was isolated using 20% acrylamide gel containing urea (8 M).

Reaction with Cyclic DNA

CDDP or MTPC and cyclic DNA B-C were added to PBS in the ratio 0.5, 1.0, and 2.0 of Pt to DNA, respectively, and incubated at 37 °C overnight. The reaction mixture was separated using 20% acrylamide gel containing urea (8 M) to analyze the interaction between the complex and cyclic DNA.

MtDNA Damage and Transcription Inhibition

A549 cells were seeded into 6-well plates at a density of 2×10^5 cells per well, cultured at 37 °C for 24 h, and treated with CDDP and MTPC (3 μM) for 24 h,

respectively. DNA and RNA were extracted and their quantity and purity were measured by Nanodrop 1000. The RT-qPCR reactions were performed with the CFX Connect^{TM} Real-Time System (BIO-RAD, USA) using a SYBR-Green kit (BIORAD, USA). All primers were synthesized by GENEray Biotechnology (Shanghai). The primer sequences are listed in Tables S3 and S4.

Cellular total DNA was isolated and purified using a genomic DNA mini preparation kit after treatment with the complex. The level of mtDNA damage in each region of the mitochondrial genome was quantified by comparing the relative amplification of long and short mtDNA fragments that were located in the same region, with the shorter fragments as internal normalization controls. qPCR was performed using the total DNA (5 ng) at 95 °C for 10 min, followed by 39 cycles at 95 °C for 10 s, 60 °C for 10 s, and 72 °C for 50 s. The mtDNA damage was evaluated on the amplification efficiency alteration calculated by the $2^{-\Delta\Delta Ct}$ method.[3]

RNA was isolated using total RNA extraction kits (Life Technologies, USA), and ~1 μg of total RNA was reverse-transcribed to cDNA using an iScript cDNA synthesis kit (BIO-RAD, USA) according to the manufacturer's instruction. The expression of 13 mitochondrial code genome proteins was analyzed by RT-qPCR. The amplification program was 30 s at 95 °C, followed by 49 cycles of 5 s at 95 °C and 5 s at 60 °C. At the end of amplification, the specificity of the gene was assessed by a melting curve between 65 and 95 °C. The results were calculated according to the $2^{-\Delta\Delta Ct}$ method. The effect of MTPC on mitochondrial copy number was determined similarly.

Mitochondrial Bioenergetics

The oxygen consumption rate (OCR) was determined using Seahorse XF^{e}24 cell MitoStress test kit (Seahorse Bioscience, Massachusetts, USA). Breifly, cells at a density of 5×10^3 per well were seeded in 24-well plates, followed by culturing at 37 °C for 18 h. The growth medium was replaced by medium supplemented with CDDP and MTPC (3 μM), respectively, and incubated at 37 °C for 24 h. XF assay medium (Seahorse Bioscience) containing glucose (25 mM) and pyruvate (2 mM) or glutamine (2 mM) was added to the wells along with each complex to maintain a stimulating

environment. The cells were equilibrated at 37°C in a CO_2-free incubator for 1 h. During the measurement, oligomycin, FCCP, and rotenone plus antimycin A (1 μM each) were injected consecutively every 24 min. In addition, A549 cells were seeded in a 6-well plate at a density of 2×10^5 cells per well and incubated overnight. The cells were treated with CDDP or MTPC for 24 h, the medium was removed and the cells were washed with PBS. ATP generation was measured according to the enhanced ATP assay kit (Beyotime). Results were normalized to the total protein level in the cell lysate assessed in each well by the BCA assay (Bio-Rad, Hercules, CA, USA).

Mitochondrial Morphology

A549 cells were inoculated in 6 cm plates and cultured overnight at 37 °C. The cells were incubated with the complex (3 μM) for 24 h and harvested. The cells were fixed with glutaraldehyde (2.5%) at 4 °C overnight, washed with PBS, and further fixed with OsO_4 (1%). The samples were dehydrated sequentially using the solutions of acetone (50%, 75%, 90% and 100%) prior to impregnation in increasing concentrations (25%, 50%, 75% and 100%) of resin in acetone over 24 h. The cells were collected on copper grids and TEM images were taken by the Hitachi TEM system.

Mitochondrial Membrane Potential

The effect of MTPC and CDDP on the mitochondrial membrane potential ($\Delta\Psi_m$) was tested using 5,5,6,6′-tetrachloro-1,1′,3,3′-tetraethyl-imidacarbocyanine iodide (JC-1) molecular probe. Briefly, A549 cells were seeded in a black 96-well plates with a density of 4×10^3 cells per well and cultured at a confluence of 70–80% for 24 h. The cells were treated with CDDP or MTPC at different concentrations and 37 °C for 24 h, and washed with PBS and stained with JC-1 (5 μg mL^{-1}) at 37 °C in the dark for 20 min. The cells were washed twice with PBS and measured by Thermo Scientific Microplate Reader.

Release of MtDNA

A549 cells were seeded in a glass-bottom cell culture dish (φ 2 mm, NEST) and incubated at 37 °C for 18 h. CDDP or MTPC (3 μM) was added to the cells and incubated for 24 h. The cells were stained with MitoTracker Deep Red (MTDR) for 30

min at 37 °C and Peko Green for 1 h at 37 °C, respectively. The wells were then counterstained with DAPI for 10 min and washed twice with PBS, and fluorescent images were taken by confocal laser scanning microscope (Zeiss LSM 710). MTDR: λ_{ex} = 633 nm, λ_{em} = 660 ± 20 nm; Peko Green: λ_{ex} = 490 nm, λ_{em} = 525 ± 20 nm; DAPI: λ_{ex} = 340 nm, λ_{em} = 488 nm.

Measurement of Cytosolic MtDNA

A549 cells were treated with CDDP or MTPC (3 μM) for 24 h and divided into 2 equal aliquots. One was used to extract total DNA by animal DNA isolation kit, and the other was used to extract cytosolic DNA by ReadiPrep™ mitochondrial/cytoplasmic fractionation kit. RT-qPCR was preformed using a CFX Connect^{TM} Real-Time System (BIO-RAD, USA) using a SYBR-Green kit (BIORAD, USA). The primers of ND1, ND4 and MTCO1 were synthesized by Generay Biotechnology (Shanghai) and the sequences were listed in Table S3.

Expression of Proteins

Cells were treated with CDDP or MTPC (3 or 5 μM), collected and washed twice with ice-cold PBS. The cell pellets were lysed in RIPA lysis buffer (50–100 μL, Beyotime) with protease inhibitor cocktail (Sigma), phenylmethanesulfonyl fluoride (PMSF, 1 mM) and dithiothreitol (DTT, 5 mM) on ice for several min. The lysate was obtained by centrifugation at 12,000 × g for 10 min at 4 °C. Total protein concentration was determined by Bradford assay. Subsequently, total protein (40–80 μg) was separated by sodium dodecyl sulfate polyacrylamide gel electrophoresis (10–20%) at 100 V for 1.5 h and transferred to poly(vinylidene difluoride) membranes (Millipore, Billerica, MA). Primary antibodies (LC3B, p62, mtTFA, cGAS, STING, p-TBK, p-IRF3, IRF3, IFN-β, GAPDH, β-actin, BAK, BAX, POLG) and secondary antibody of goat antirabbit IgG H&L (HRP) were used to perform the western blotting assay. The proteins were visualized by enhanced chemiluminescence kits (Millipore Corporation). The protein expression levels were quantitatively analyzed by Image J software.

Detection of 8-OHdG

A549 cells were seeded in a glass-bottom cell culture dish (φ 2 mm, NEST) and

incubated at 37 °C for 18 h. CDDP or MTPC (3 μM) was added to the cells and incubated for 24 h. The cells were stained with Mito-Tracker Deep Red for 30 min at 37 °C and then fixed with 4% formaldehyde, followed by treatment with 0.1% Triton X-100 in PBS for 5 min, and blocked with 5% bovine serum albumin in PBS containing 0.1% Tween-20 for 30 min. The cells were immunoblotted with primary antibody anti-8-OHdG antibody (1:500, MCE) at 4 °C for 12 h, and washed thrice in PBS before incubating in the dark with a FITC-labeled secondary antibody goat anti-rabbit IgG H&L (1:500, Abcam) for 60 min. After washing with PBS containing 0.1% Tween-20 for 3 times, the cells were counterstained with DAPI for 10 min. The wells were emptied and washed with PBS twice, and fluorescent images were taken by confocal laser scanning microscope (Zeiss LSM 710). Mito-Tracker Deep Red: λ_{ex} = 644 nm, λ_{em} = 665; DAPI: λ_{ex} = 340 nm, λ_{em} = 488 nm.

2. Supplemental Figures and Tables

Table S1. IC_{50} (μM) of MTPC and CDDP against A549 and HK-2 cell lines at 48 h, respectively. Data are the average of three measurements.

Cell line	A549	HK-2
MTPC	3.09 ± 0.78	22.41 ± 1.46
CDDP	2.40 ± 0.60	4.96 ± 0.71

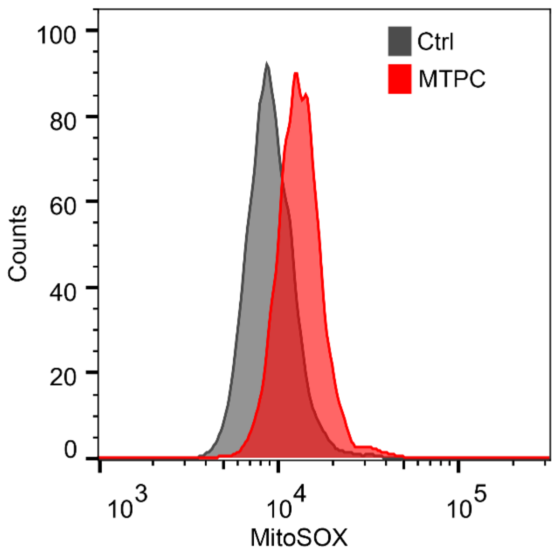

Figure S1. Flow cytometric analysis of mitochondrial ROS in A549 cells treated with MTPC (3 μM) for 24 h determined by a fluorescent probe MitoSOX at 580 nm.

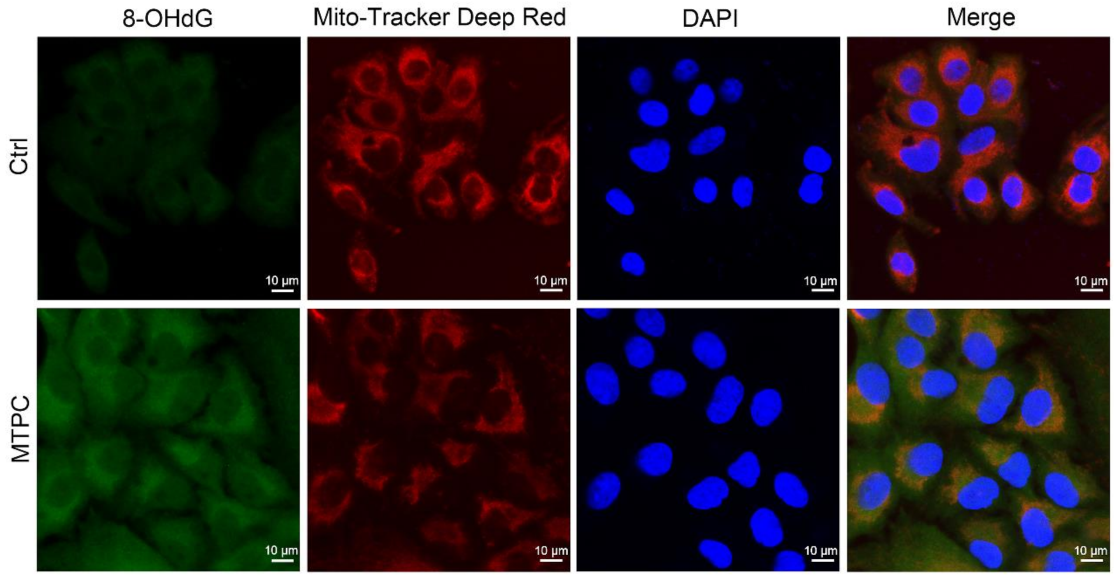

Figure S2. Oxidative damage of mtDNA in A549 cells induced by MTPC (3 μM) at 24 h and detected by immunofluorescence staining with an anti-8-OHdG antibody (green), Mito-Tracker Deep Red (red), and DAPI (cell nuclei, blue).

Table S2. Primers used in the synthesis of circular DNA.

Primer	Sequence
B-C	GGGAAGGGACTGAGAATAAG
B-sp	GTCCCTTCCCGTCTCTTCTGTCCCGGTCTCTGCTCTCTCTTATTCTCA

Table S3. Primers used in the qPCR assay.

Primer	Sequence
AS1.F	CCCTAACACCAGCCTAACCA
AS1.R	AAAGTGCATACCGCCAAAAG
BS1.F	CATGCCCATCGTCCTAGAAT
BS1.R	ACGGGCCCTATTTCAAAGAT
CS1.F	TCCAACTCATGAGACCCACA
CS1.R	TGAGGCTTGGATTAGCGTTT
DS1.F	ACTACAACCCTTCGCTGACG
DS1.R	GCGGTGATGTAGAGGGTGAT
AL1.F	CTGTTCTTTCATGGGGAAGC
AL1.R	AAAGTGCATACCGCCAAAAG
BL1.F	CATGCCCATCGTCCTAGAAT
BL1.R	TGTTGTCGTGCAGGTAGAGG
CL1.F	CACACGAGAAAACACCCTCA
CL1.R	CTATGGCTGAGGGGAGTCAG
DL1.F	CCCTTCGCCCTATTCTTCAT
DL1.R	GCGTAGCTGGGTTTGGTTTA
BS1F	CATGCCCATCGTCCTAGAAT
BS1R	ACGGGCCCTATTTCAAAGAT
CHIP9F	CACCTACCTCCCTCACCAAA
CHIP9R	GGGATCAATAGAGGGGGAAA
LC3-F	GTGAATTGGGCTGTGAGTGT
LC3-R	AGCCAAAGGTGCATTTCGTA
MT-ND1F	GGCTATATACAACTACGCAAAGGC
MT-ND1R	GGTAGATGTGGCGGGTTTTAGG
MT-ND4F	CCCTCGTAGTAACAGCCATTCTC
MT-ND4R	CGACTGTGAGTGCGTTCGTAGT
MT-CO1F	CTTTTCACCGTAGGTGGCCT
MT-CO1R	AGTGGAAGTGGGCTACAACG
GAPDH-F	GTCTCCTCTGACTTCAACAGCG
GAPDH-R	ACCACCCTGTTGCTGTAGCCAA

Table S4. Primers used in the RT-qPCR assay.

Gene	Primer
MT-ND1F	ATATGAAGTCACCCTAGCCAT
MT-ND1R	CTGAGACTAGTTCGGACTCCC
MT-ND2F	CGGACAATGAACCATAACCAA
MT-ND2R	GTTTAATCCACCTCAACTGCC
MT-ND3F	GCCCTACAAACAACTAACCTG
MT-ND3R	ATTCGGTTCAGTCTAATCCTT

MT-ND4F	TCTGTGCTAGTAACCACGTTC
MT-ND4R	AAAACCCGGTAATGATGTCG
MT-ND4LF	ACTAGTATATCGCTCACACC
MT-ND4LR	CTAGTATGGCAATAGGCACA
MT-ND5F	CTTACCACCCTCGTTAACCC
MT-ND5R	ATAACTTCTTGGTCTAGGCACA
MT-ND6F	ATATACTACAGCGATGGCTA
MT-ND6R	AATCCTACCTCCATCGCTA
MT-CYBF	TTATTGACTCCTAGCCGCAGA
MT-CYBR	TAGTACGGATGCTACTTGTCCA
MT-CO1F	AATAGGAGCTGTATTTGCCAT
MT-CO1R	AGAAAGTTAGATTTACGCCGAT
MT-CO2F	CTTTACATAACAGACGAGGTCA
MT-CO2R	TTGAAGATTAGTCCGCCGTA
MT-CO3F	CCACTCCTAAACACATCCGTA
MT-CO3R	GCCAATAATGACGTGAAGTCC
MT-ATP6F	CAACACTAAAGGACGAACCTG
MT-ATP6R	TTAATCTTAGAGCGAAAGCCTA
MT-ATP8F	TGCCCCAACTAAATACTACCG
MT-ATP8R	ATGAATGAAGCGAACAGAT
GAPDH-F	GGAGCGAGATCCCTCCAAAAT
GAPDH-R	GGCTGTTGTCATACTTCTCATGG

References

(1) Wu, S. N.; Wang, X. Y.; He, Y. F.; Zhu, Z. Z.; Zhu, C. C.; Guo, Z. J. A Monofunctional Trinuclear Platinum Complex with Steric Hindrance Demonstrates Strong Cytotoxicity Against Tumor Cells. *J. Inorg. Biochem.* **2014**, *139*, 77–84.

(2) Jin, S. X.; Muhammad, N.; Sun, Y. W.; Tan, Y. H.; Yuan, H.; Song, D. F.; Guo, Z. J.; Wang, X. Y. Multispecific Platinum(IV) Complex Deters Breast Cancer via Interposing Inflammation and Immunosuppression as an Inhibitor of COX-2 and PD-L1. *Angew. Chem. Int. Ed.* **2020**, *59*, 23313–23321.

(3) Guo, Y.; He, Y. F.; Wu, S. D.; Zhang, S. R.; Song, D. F.; Zhu, Z. Z.; Guo, Z. J.; Wang, X. Y. Enhancing Cytotoxicity of a Monofunctional Platinum Complex via a Dual-DNA-Damage Approach. *Inorg. Chem.* **2019**, *58*, 13150−13160.
